# Supplementary material for: Learning sparse log-ratios for high-throughput sequencing data
Source: Bioinformatics. 2021 Sep 8;38(1):157–63. doi: 10.1093/bioinformatics/btab645 (PMC8696089; doi:10.1093/bioinformatics/btab645)
Supplement: btab645_Supplementary_Data [file btab645_supplementary_data.zip › supp.pdf]

Supplementary Material for: Learning Sparse Log-Ratios for High-Throughput Sequencing Data

Elliott Gordon-Rodriguez[1*], Thomas P. Quinn[2], and John P. Cunningham[1]

[1]Department of Statistics, Columbia University, New York, USA
[2]Applied Artificial Intelligence Institute (A2I2), Deakin University, Geelong, Australia
*eg2912@columbia.edu

A Software Package

We provide open source implementations of CoDaCoRe as R[1] and Python[2] packages. Deploying these on novel datasets is straightforward:

```
devtools::install_github("egr95/R-codacore")
model <- codacore(x, y)
print(model)
plot(model)
```

Detailed documentation and examples may be found at the *CoDaCoRe Guide*.[3]

B Other Related Work

CoDa methodology has also recently attracted interest from the machine learning community (Tolosana-Delgado *et al.*, 2019; Quinn *et al.*, 2020; Gordon-Rodriguez *et al.*, 2020a,b; Templ, 2020). Relevant to us is *DeepCoDA* (Quinn *et al.*, 2020), which combines self-explaining neural networks with log-ratio transformed features. In particular, DeepCoDA learns a set of *log-contrasts*, in which the numerator and denominator are defined as *unequally weighted* geometric averages of components Egozcue and Pawlowsky-Glahn (2016). As a result of this weighting, DeepCoDA loses much of the interpretability and intuitive appeal of balances (or amalgamations), which is exacerbated by its lack of sparsity. Moreover, like most deep architectures, DeepCoDA is sensitive to initialization and optimization hyperparameters

[1]https://github.com/egr95/R-codacore
[2]https://github.com/egr95/py-codacore
[3]https://egr95.github.io/R-codacore/guide.html

Table 1: Qualitative comparison of learning algorithms, ordered from most sparse (top) to least (bottom). CoDaCoRe is the only method that performs on all of our criteria. See Table 1 for a corresponding quantitative comparison.

	Scalability	Interpretability	Sparsity	Accuracy
**CoDaCoRe (ours)**	+	+	+	+
Pairwise log-ratios (Greenacre, 2019b)	−	+	+	−
Selbal (Rivera-Pinto *et al.*, 2018)	−	+	+	·
Lasso	+	·	+	−
Coda-lasso (Lu *et al.*, 2019)	−	·	·	·
Amalgam (Quinn and Erb, 2020)	−	+	−	·
DeepCoDA (Quinn *et al.*, 2020)	·	·	−	·
CLR-lasso (Susin *et al.*, 2020)	+	−	−	+
Black-box (e.g., Random Forest, XGB)	+	−	−	+

(which limits its ease of use) and is susceptible to overfitting (which can further compromise interpretability of the model).

The special case of a linear log-contrast model has been referred to as *Coda-lasso*, and was separately proposed by Lu *et al.* (2019). While Coda-lasso scales better than selbal, it has been found to perform worse in terms of predictive accuracy (Susin *et al.*, 2020). More importantly, Coda-lasso is still prohibitively slow on the high-dimensional HTS data that we wish to consider. Last, we highlight another common set of features that are also a special case of log-contrasts: *centered-log-ratios* (CLR), where each input variable is divided by the geometric mean of *all* input variables (Aitchison, 1982). Models using these features, such as CLR-lasso (Susin *et al.*, 2020), can be accurate and computationally efficient, however they are inherently not sparse and are difficult to interpret scientifically (Greenacre, 2019a).

C Additional Methodological Detail

Table 1 provides a summarized comparison of the relative merits of CoDaCoRe and its competitors. Algorithm 1 provides a summary of the CoDaCoRe learning algorithm.

C.1 Continuous Relaxation

We optimized Eq. 8 from the main text using gradient descent with the following specifications:

- **Initialization.** We initialized the weights parameter $\mathbf{w}$ at zero, which corresponds to assigning no prior "preference" to one input variable over any another. As for the intercept parameter α, initialization requires some care, particularly in order to

Algorithm 1 CoDaCoRe

Inputs: Training data: $(\mathbf{x}_i, y_i)_{i=1}^n$.
Initialize $\hat{g}(\mathbf{x}) = 0$.
repeat
  Initialize a new relaxation $(\mathbf{w}, \alpha, \beta)$.
  Train $(\mathbf{w}, \alpha, \beta)$ by gradient descent.
  Use cross-validation to find the optimal threshold, $\hat{t}$.
  Retrain (α, β) using $(\hat{J}^+, \hat{J}^-)$.
  Update ensemble $\hat{g}(\mathbf{x}) \leftarrow \hat{g}(\mathbf{x}) + \hat{f}(\mathbf{x})$.
until $\hat{J}^+ = \emptyset$ or $\hat{J}^- = \emptyset$.
Return $\hat{g}(\mathbf{x})$.

account for possibly imbalanced classes and for the stage-wise additive nature of our model. Namely, α should captures the central tendency of the response so that the gradients propagated to $\mathbf{w}$ capture the *differential* effect of the input variables on the response (not doing so severely hinders gradient descent). In practice, this amounts to initializing α at the estimated coefficient of a generalized linear model with nothing but an intercept (which, in the regression case, reduces to the mean of the response). Note that, from the second stage of ensembling onwards, the fitted value of the current ensemble must be incorporated into said generalized linear model as a fixed offset term. Finally, we found the initialization of β to matter less, and simply used the initial value $\beta = 0.1$ throughout.

- **Epochs.** We trained each continuous relaxation for 100 epochs. While this number of epochs was sufficient for the linear functionals we considered, we expect a higher number would be needed in order to combine our balances (or amalgamations) with nonlinear functionals.

- **Learning rate.** We used an adaptive scheme that exploits the structure of our continuous relaxation. Intuitively, we seek a learning rate that will allow $\mathbf{w}$ to explore the range of values where our relaxation has non-vanishing gradients, which approximately corresponds to $w_i \in (-5, 5)$. This interval corresponds to soft assignments approximately between $\tilde{w_i} \in (-0.99, 0.99)$; outside of this range, the sigmoid starts to saturate and gradients vanish. The question is then: how to choose a learning rate that allows $\mathbf{w}$ to explore the range $(-5, 5)$ efficiently (i.e., over a minimal number of epochs). We solve this indirectly; we pick the unique learning rate that would result in an initial gradient step that takes the maximum weight, $\max(|\mathbf{w}|)$, to the value 0.5. This learning rate can be found by simply computing one backward pass (and taking the maximum over the gradients w.r.t. $\mathbf{w}$) prior to applying any gradient steps. Note that the value of 0.5 is somewhat arbitrary, and simply represents the size of an initial step that should be "small, but significant relative to the target range of $(-5, 5)$".

- **Momentum.** We used momentum, with rate 0.9.
- **Minibatching.** We did not use minibatching, as we found it unnecessary on the datasets we considered. Nevertheless, minibatching could be beneficial, particularly for datasets with larger number of observations.

C.2 Discretization

Given a trained vector of soft assignments $\tilde{\mathbf{w}}$, typically only a small number will have converged to values close to $+1$ or -1, with most components remaining close to zero. As discussed in Section 3.3, in order to identify a balance it is sufficient to specify a threshold value t. However, different thresholds $t \in (0,1)$ can result in different balances, depending on the values of $\tilde{\mathbf{w}}$. Our implementation evaluates a set of 20 "candidate thresholds", $t \in \{t_1, \ldots, t_{20}\}$, and settles on the one that yields the best cross-validation score. Any grid of values $\{t_1, \ldots, t_{20}\} \in (0,1)$ can be used, where we write $\{t_1, \ldots, t_{20}\}$ in decreasing order. A principled choice is to "step through" the weights. Namely, start by rescaling the positive and negative components of $\tilde{\mathbf{w}}$ so that $\max \tilde{\mathbf{w}}^+ = \max \tilde{\mathbf{w}}^-$, and then set $\{t_1, \ldots, t_{20}\}$ to equal the top 20 order statistics of the set $\{|\tilde{w}_j|\}$. Thus, the first candidate t_1 will yield a simple log-ratio between two input variables (the rescaling ensure precisely two of our weights are $\geq t_1$), the second candidate t_2 will yield a log-ratio with a total of three input variables, and so forth.

Given a set of candidate thresholds $\{t_1, \ldots, t_{20}\}$, we associate a score to each of these via cross-validation. We first split the training data into 5 folds (sampled with stratification by case control (He and Ma, 2013)). Then, for each candidate threshold t_i, we fit 5 regressors of the form Eq. 3 on the 5 cross-validation training sets, we evaluate on their respective validation folds, and average the results to obtain the overall cross-validation score of t_i. Finally, we choose the largest threshold (i.e., the sparsest model) whose cross-validation score is within 1 standard error of the optimum achieved over $\{t_1, \ldots, t_{20}\}$ (with standard errors computed over the 5 cross-validation folds).

Note that we could search in this way over more than 20 candidate thresholds; the choice of the number 20 is by analogy to *selbal* (Rivera-Pinto *et al.*, 2018), where balances of up to (but no more than) 20 input variables are considered. We also found that our results were broadly insensitive to searching over more candidate thresholds; increasing this number can lead to slightly more accurate models that are slightly less sparse, and, evidently, somewhat slower to compute.

D Extensions

Our model allows for a number of extensions:

- *Unsupervised learning.* By means of a suitable unsupervised loss function, CoDaCoRe can be extended to unlabelled datasets, $\{\mathbf{x}_i\}_{i=1}^n$, as a method for identifying

log-ratios that provide a useful low-dimensional representation. Such a method would automatically provide a scalable alternative to several existing dimensionality reduction techniques for CoDa (Pawlowsky-Glahn *et al.*, 2011; Mert *et al.*, 2015; Martín-Fernández *et al.*, 2018; Martino *et al.*, 2019; Quinn *et al.*, 2021).

- *Incorporating confounders.* In addition to $(\mathbf{x}_i, y_i)_{i=1}^n$, in some applications the effect of additional (non-compositional) covariates, $\mathbf{z}_i$, is also of interest. In this case, the effect of $\mathbf{z}_i$ can be "partialled out" a priori by first regressing y_i on $\mathbf{z}_i$ alone, and using this regression as the initialization of the CoDaCoRe ensemble. Alternatively, $\mathbf{z}_i$ can also be modeled jointly in Equations 3 and 11 (e.g., by adding a linear term $\gamma \cdot \mathbf{z}_i$) (Forslund *et al.*, 2015; Noguera-Julian *et al.*, 2016; Rivera-Pinto *et al.*, 2018).

- *Nonlinear regression functions.* Our method extends naturally to nonlinear regression functions of the form $f(\mathbf{x}) = h_\theta(B(\mathbf{x}; J^+, J^-))$, where h_θ is a parameterized differentiable family, including neural networks (Morton *et al.*, 2019; Quinn *et al.*, 2020).

- *Applications to non-compositional data.* Aggregations of parts can be useful outside the realm of CoDa; for example, an amalgamation applied to a categorical variable with many levels represents a grouping of the categories (Bondell and Reich, 2009; Gertheiss and Tutz, 2010; Tutz and Gertheiss, 2016).

E Datasets

Table 4 provides further details on the 25 datasets used in Section 4 of the the main text. Note also that zero-replacement is necessary prior to applying log-ratio transformations, a standard pre-processing step in the field of CoDa (Martín-Fernández *et al.*, 2000; Aitchison *et al.*, 2003; Martín-Fernández *et al.*, 2012). When necessary, we carried out zero-replacement by simply adding one unit to all counts prior to normalization.

F Additional Experimental Detail

Tables 5, 6, 7, 9 show our evaluation metrics on each individual dataset for a selection of our models. Table 1 from the main text summarizes these four tables by averaging over all datasets. Figure 1 shows the same heatmap as Figure 2 from the main text, but with amalgamations as well as balances. Differences between the sets selected by CoDaCoRe with balances vs. CoDaCoRe with amalgamations can be explained by differences in how the geometric mean vs. summation operations impact the log-ratio. The geometric mean, being more sensitive to small numbers, is more affected by the presence of rarer bacteria species like Dialister and Roseburia (as compared with the more common bacteria species like Haemophilus and Faecalibacterium).

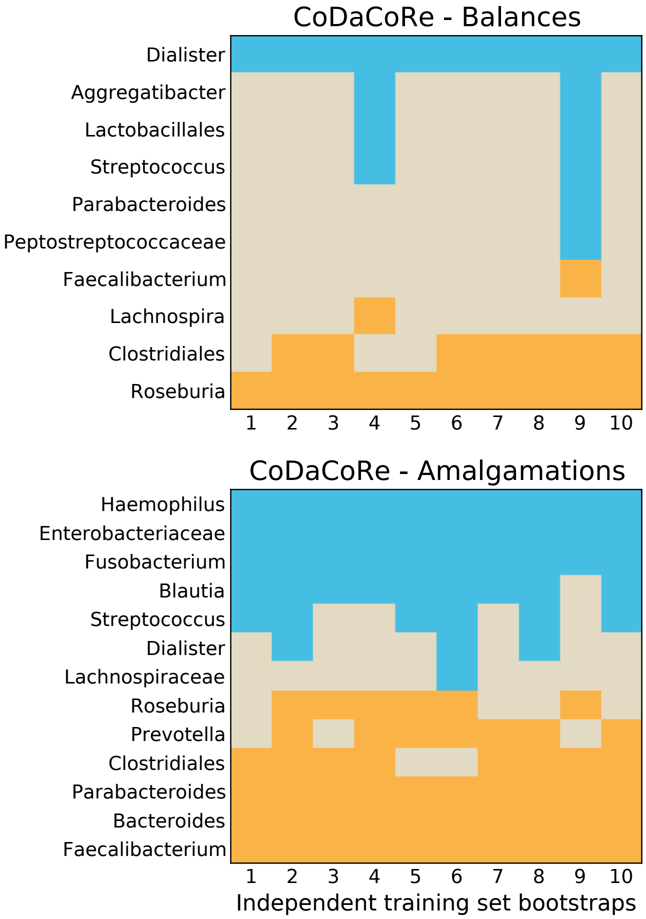

Figure 1: CoDaCoRe variable selection for the first (most explanatory) log-ratio on the Crohn disease data (Rivera-Pinto *et al.*, 2018). For each of 10 independent bootstraps of the training set (80% of the data randomly sampled with stratification by case-control), we show which variables are selected in the numerator (blue) and denominator (orange) of the log-ratio. Both versions of CoDaCoRe, with balances (top) or amalgamations (bottom), learn remarkably consistent log-ratios across independent training sets. Differences between the sets selected by CoDaCoRe with balances vs. CoDaCoRe with amalgamations can be explained by differences in how the geometric mean vs. summation operations impact the log-ratio. The geometric mean, being more sensitive to small numbers, is more affected by the presence of rarer bacteria species like Dialister and Roseburia (as compared with the more common bacteria species like Haemophilus and Faecalibacterium).

F.1 Critical Difference Diagrams

Critical difference (CD) diagrams are a robust non-parametric tool for the comparison of multiple classifiers over multiple datasets (Demšar, 2006). For a given success metric (e.g., prediction error), these diagrams display the average rank of each method, taken by sorting the methods from best to worst on each individual dataset. In addition, horizontal lines link together the methods whose ranks were not significantly different from one another, for the metric under consideration. We measure significance at the 5% level, using the Wilcoxon signed rank test (Wilcoxon, 1992) with Holm's alpha correction (Holm, 1979).[4] Figure 2 shows the critical difference diagrams evaluated over our 6 interpretable CoDa methods: selbal, amalgam, pairwise log-ratios, Coda-lasso, and CoDaCoRe (with default parameters). We show a separate diagram for each of our 5 performance metrics: runtime, sparsity (proportion of variables active), classification accuracy, AUC, and F1 score (all measured on the held-out test set). The results are broadly consistent with Table 1 from the main text. Namely, CoDaCoRe performs highly across all of our criteria; it is (by far) the most computationally efficient, as well as the sparsest (tied with selbal and pairwise log-ratios), and the most predictive (tied with amalgam in terms of accuracy, and tied with most other competitors in AUC and F1 score). Note that while CoDaCoRe (with default parameters) scored equally well on AUC and F1 relative to other methods, when running CoDaCoRe with reduced regularization ($\lambda = 0$), it did become significantly more predictive than any of these methods (at the expense of some sparsity). Figure 3 corroborates these observations, showing the direct pairwise comparisons between each method, separately for each evaluation metric.

F.1.1 Ablations

While CoDaCoRe clearly outperforms its competitors in terms of runtime and sparsity, the differences in predictive accuracy are more nuanced, and often not statistically significant. One may therefore ask whether there exist classes of problems on which CoDaCoRe will tend to out- or under-perform competing methods. To answer this question, we regress the rank scores from the previous section against (a) number of observations, (b) number of input variables, (c) class imbalance, and (d) a proxy for the signal-to-noise ratio. Namely, for each of our 25 datasets we first rank the predictive accuracy score of CoDaCoRe relative to the 5 other methods (lower rank is better), resulting in 25 datapoints. We then plot these separately against each of (a), (b), (c), and (d), together with the corresponding trendline (obtained from 4 separate univariate regressions). The class imbalance (c) is shown as a number between 0.5 and 1, representing the proportion of datapoints in the majority class (i.e., the baseline accuracy). The signal-to-noise ratio (d) was approximated by taking the out-of-sample accuracy of the Random Forest model, and subtracting the

[4] We used the implementation of Fawaz *et al.* (2019) available at https://github.com/hfawaz/cd-diagram

baseline accuracy (majority vote).[5] The results are shown in Figure 4. While our collection of 25 datasets may not be sufficient to draw firm conclusions, it seems that CoDaCoRe performs consistently across high- and low-dimensional datasets, as well as across the high- and low-signal datasets. Interestingly, CoDaCoRe tends to outperform most strongly on the datasets where the number of observations is small, possibly indicating superior sample efficiency over competing methods. Plotting a similar ablation against the ratio n/p shows a very similar effect, and is omitted for brevity. CoDaCoRe also tends to rank slightly higher on datasets with lower class imbalance, indicating that our algorithm may benefit from class rebalancing, a line that is left to future work.

F.2 Implementation Detail for Competing Methods

The competing methods used in our experiments were ran as follows:

- Selbal (Rivera-Pinto *et al.*, 2018) was run using the `selbal.cv` function from the implementation of the original authors, with default parameters.[6]
- Pairwise log-ratios (Greenacre, 2019b) was run using the `pra` function from the `propr` package (available on CRAN), with `ndim=10`.
- Lasso was run using `cv.glmnet` from the `glmnet` package, with default parameters. The regularization strength was selected using the 1-standard-error rule.
- Coda-lasso (Lu *et al.*, 2019) was run using the implementation of Susin *et al.* (2020).[7] In particular, we combined the `coda_logistic_lasso` function with cross-validation (again, with the 1-standard error rule), to select the regularization strength.
- Amalgam (Quinn and Erb, 2020) was run using the `amalgam` function from the implementation of the original authors.[8] Since our work compares log-ratio selection methods, we used the parameter `asSLR=TRUE`, which in turn implied we had to change the `numAmal` parameter from its default value of 3 to 6, since an even number is required (for the numerators and denominators of the SLR, respectively).
- DeepCoDA (Quinn *et al.*, 2020) was adapted from the implementation of the original authors (in order to run their Tensorflow code in R).[9]
- CLR-lasso (Susin *et al.*, 2020) was implemented by applying `cv.glmnet` to CLR-transformed data, again, choosing the regularization parameter with the 1-standard-error rule.

[5] Note this metric is intended as a rough proxy and we do not claim to accurately estimate the signal-to-noise ratio of any of our datasets.

[6] https://github.com/malucalle/selbal

[7] https://github.com/malucalle/CoDA-Penalized-Regression

[8] https://github.com/tpq/amalgam

[9] https://github.com/nphdang/DeepCoDA

- Random Forest was run with the `tuneRF` function from the `randomForest` package (available on CRAN), with `doBest=TRUE` (and default parameters otherwise). This is in order to select the best value of the `mtry` parameter with respect to out-of-bag error.
- Log-ratio lasso (Bates and Tibshirani, 2019) was run using the `cv_two_stage` function from the `logratiolasso` package written by the original authors, with default parameters.[10]

G Simulation Study

G.1 Low-Dimensions

Our first simulated dataset is taken directly from Susin *et al.* (2020). The data-generating process starts with the observed HTS counts of the Crohn's Disease data from Gevers *et al.* (2014). These data consist of $n = 975$ samples and $p = 48$ genera. For each simulation scenario, the abundance table was obtained by randomly selecting k "active" variables and $\tilde{k}$ "inactive" variables from the p columns, and discarding the rest. The simulation parameters were varied over $k \in \{3, 5, 10\}$ and $\tilde{k} \in \{10, 20, 30, 40\}$. For each such combination, 10 independent simulations were generated, and a balance was defined over the k active variables. For example, in the case $k = 3$, the first variable was used for the numerator, and the other 2 for the denominator.[11] In turn, a binary response was simulated for each datapoint by drawing a Bernoulli random variable, with probability of success equal to the sigmoid of the computed balance. In all, this gave a total of $3 \times 4 \times 10 = 120$ simulated datasets, on each of which we fitted CoDaCoRe (with default parameters), selbal (Rivera-Pinto *et al.*, 2018), and coda-lasso (Lu *et al.*, 2019). The results are shown in figure 5. Perhaps unsurprisingly (given the large number of samples relative to the number of input variables), all three methods are able to recover the true active variables in the data-generating process, across all 120 datasets. Both CoDaCoRe and selbal were able to do so while maintaining a very low false positive rate, meaning that they almost never selected any of the variables that were not part of the true data-generating process. On the other hand, coda-lasso selected a large amount of false positive variables on many of the datasets, in spite of having tuned its regularization parameter (i.e., L_1 penalty) using the sparsity-inducing 1-standard-error rule on each individual dataset.

G.1.1 Heterogeneous balances

We additionally evaluate the ability of CoDaCoRe to recover *multiple* log-ratios, in the more challenging setting where the true data-generating process is driven by two balances with

[10] https://github.com/stephenbates19/logratiolasso

[11] Note that in the $k = 10$ case, Susin *et al.* (2020) used an imbalanced log-linear combination, which we changed to a regular balance for the purposes of our study.

Table 2: Variable selection performance for CoDaCoRe, selbal, and coda-lasso for simulated datasets with two generative balances of heterogeneous effect sizes. In all, $4 \times 10 = 40$ datasets were used to produce this table. For CoDaCoRe and selbal, we can distinguish between the first and the second learned log-ratios, whereas coda-lasso learns a single log-linear combination for the entire dataset.

	TPR	FPR
CoDaCoRe (first learned balance)	1.00 ± 0.00	0.01 ± 0.02
CoDaCoRe (second learned balance)	0.61 ± 0.29	0.08 ± 0.11
selbal (first learned balance)	1.00 ± 0.00	0.00 ± 0.00
selbal (second learned balance)	0.00 ± 0.00	0.13 ± 0.07
coda-lasso	1.00 ± 0.00	0.38 ± 0.11

heterogeneous effect sizes. To do so, we again follow the simulation scheme of Susin *et al.* (2020), as per the previous section, except for now taking $k = 6$ active variables. Of the 6 variables active, 3 will again form one balance (with the first variable in the numerator and the other 2 in the denominator), and the other 3 will form a "secondary" balance (also with 1 numerator and 2 denominator variables). The effect size of the secondary balance is set to 1/3 of that of the first balance. In other words, the binary response is simulated by computing a linear combination of two balances, the sigmoid of which equals the probability of success. We repeat the process over the same previous values of $\tilde{k} \in \{10, 20, 30, 40\}$, with 10 independent draws for each. On the resulting 40 datasets, we fit CoDaCoRe, selbal and coda-lasso. CoDaCoRe will automatically find two log-ratios (provided there exists sufficient signal in the data), but to accomplish the same with selbal, we had to run the algorithm twice, feeding the first balance as a covariate in the second pass. On the other hand, coda-lasso does not establish balances per se, but rather a log-linear combination where each input variable has a different weight. While it is not obvious in general how these may be combined to produce bona fide balances, in this case we simply measured the ability of coda-lasso to recover all 6 active variables. Note that this is a substantially less stringent criteria than was demanded of CoDaCoRe or selbal, where we compared the first learned balance specifically to the 3 variables from the primary (true) balance, and the second learned balance to the 3 variables from the secondary (true) balance. The results are shown in Table 2. CoDaCoRe is the only method that shows appreciable performance at recovering the secondary balance, typically selecting at least 2 of its 3 true constituents, while keeping a low false positive rate (8%, on average). Selbal is unable to identify the secondary balance in this heterogeneous setting, while coda-lasso does identify all the relevant variables, but with a problematic false positive rate of 38%, on average (and without specifying how the selected variables may be grouped into balances).

G.2 High-Dimensions

Next, we consider a collection of purely simulated datasets of arbitrary sizes, which will allow us to evaluate the variable selection capabilities of our methods across a wide range of input dimensions. To simulate the HTS count data of size $n \times p$, we start by drawing $\alpha \sim \text{Dirichlet}(1/\log(p), \ldots, 1/\log(p))$.[12] Then, for each datapoint $i = 1, \ldots, n$, we draw $p_i \sim \text{Dirichlet}(\alpha)$ and $n_i \sim \text{Poisson}(1000 \cdot n)$, and in turn $x_i \sim \text{Multinomial}(n_i, p_i)$. In addition, we corrupt a randomly selected 0.1% of the entries of each x_i with random Poisson(1000) noise. A balance or a summed-log-ratio is then constructed by taking $k/2$ variables in the numerator and $k/2$ variables in the denominator, and its sigmoid defines the probability of success of a Bernoulli binary response. For each $p \in \{100, 300, 1\,000, 3\,000, 10\,000\}$, and $k \in \{2, 4, 8, 16, 32\}$, we simulate 10 independent datasets, resulting in $5 \times 5 \times 10 = 250$ datasets. For each of these 250 datasets, we generate two versions, one where the response is simulated using balances, and one where the response is simulated using summed-log-ratios. We take $n = 200$ throughout. On the datasets simulated using balances, we compare CoDaCoRe (with default parameters) against selbal and coda-lasso, as shown in Figure 6. On those simulated using summed-log-ratios, we compare CoDaCoRe (with amalgamations) against amalgam (Quinn and Erb, 2020), as shown in Figure 7. Overall, we find that CoDaCoRe performs competitively, selecting most of the relevant input variables even on challenging high-dimensional datasets, all while maintaining low false positive rates. CoDaCoRe recovered the true balance slightly more accurately than selbal and coda-lasso, although with slightly worse false positive rates. The outperformance of CoDaCoRe was most pronounced in datasets with high p or high k. Importantly, selbal failed to terminate (within 100 hours) on the high-dimensional datasets; CoDaCoRe ran in a few seconds on all datasets. Likewise, in the summed-log-ratio case, CoDaCoRe clearly outperformed amalgam, with slightly worse true positive rates but dramatically better false positive rates across the board. Importantly, amalgam also failed to terminate (within 100 hours) on the high-dimensional datasets, in which CoDaCoRe was still able to run in a matter of seconds.

The outperformance of CoDaCoRe on datasets with high k showcases one of the strengths of our gradient descent procedure; since balances are learned "jointly" over all input variables, we are able to more accurately recover balances that involve a larger number of input variables (a hard problem in general). This is in contrast to the stepwise procedure used in selbal, which is more effective at identifying balances that involve a small number of input variables (i.e., those that require a small number of correct "steps" from initialization), but struggles to recover balances over many input variables (since they require a large number of correct "steps"). As the number of steps required to find a balance increases, the likelihood of making an error along the way also increases. This issue does not afflict CoDaCoRe, since gradient descent computes the contribution of each input variable to the learned balance *jointly*, "pushing" the most relevant ones towards being selected in the

[12] The parameter $1/\log(p)$ was chosen to ensure the count table obtained downstream is sparse and heavy-tailed.

discretization step downstream.

References

Aitchison, J. (1982). The statistical analysis of compositional data. *Journal of the Royal Statistical Society: Series B (Methodological)*, **44**(2), 139–160.

Aitchison, J., Kay, J. W., *et al.* (2003). Possible solution of some essential zero problems in compositional data analysis.

Bates, S. and Tibshirani, R. (2019). Log-ratio lasso: Scalable, sparse estimation for log-ratio models. *Biometrics*, **75**(2), 613–624.

Bondell, H. D. and Reich, B. J. (2009). Simultaneous factor selection and collapsing levels in anova. *Biometrics*, **65**(1), 169–177.

Demšar, J. (2006). Statistical comparisons of classifiers over multiple data sets. *The Journal of Machine Learning Research*, **7**, 1–30.

Egozcue, J. J. and Pawlowsky-Glahn, V. (2016). Changing the reference measure in the simplex and its weighting effects. *Austrian Journal of Statistics*, **45**(4), 25–44.

Fawaz, H. I., Forestier, G., Weber, J., Idoumghar, L., and Muller, P.-A. (2019). Deep learning for time series classification: a review. *Data mining and knowledge discovery*, **33**(4), 917–963.

Forslund, K., Hildebrand, F., Nielsen, T., Falony, G., Le Chatelier, E., Sunagawa, S., Prifti, E., Vieira-Silva, S., Gudmundsdottir, V., Pedersen, H. K., *et al.* (2015). Disentangling type 2 diabetes and metformin treatment signatures in the human gut microbiota. *Nature*, **528**(7581), 262–266.

Gertheiss, J. and Tutz, G. (2010). Sparse modeling of categorial explanatory variables. *The Annals of Applied Statistics*, pages 2150–2180.

Gevers, D., Kugathasan, S., Denson, L. A., Vázquez-Baeza, Y., Van Treuren, W., Ren, B., Schwager, E., Knights, D., Song, S. J., Yassour, M., *et al.* (2014). The treatment-naive microbiome in new-onset crohn's disease. *Cell host & microbe*, **15**(3), 382–392.

Gordon-Rodriguez, E., Loaiza-Ganem, G., and Cunningham, J. (2020a). The continuous categorical: a novel simplex-valued exponential family. In *International Conference on Machine Learning*, pages 3637–3647. PMLR.

Gordon-Rodriguez, E., Loaiza-Ganem, G., Pleiss, G., and Cunningham, J. P. (2020b). Uses and abuses of the cross-entropy loss: Case studies in modern deep learning. In *Proceedings on "I Can't Believe It's Not Better!" at NeurIPS Workshops*, volume 137 of *Proceedings of Machine Learning Research*, pages 1–10. PMLR.

Greenacre, M. (2019a). Comments on: Compositional data: the sample space and its structure. *TEST*, **28**(3), 644–652.

Greenacre, M. (2019b). Variable selection in compositional data analysis using pairwise logratios. *Mathematical Geosciences*, **51**(5), 649–682.

He, H. and Ma, Y. (2013). Imbalanced learning: foundations, algorithms, and applications.

Holm, S. (1979). A simple sequentially rejective multiple test procedure. *Scandinavian journal of statistics*, pages 65–70.

Lu, J., Shi, P., and Li, H. (2019). Generalized linear models with linear constraints for microbiome compositional data. *Biometrics*, **75**(1), 235–244.

Martín-Fernández, J., Barceló-Vidal, C., and Pawlowsky-Glahn, V. (2000). Zero replacement in compositional data sets. In *Data analysis, classification, and related methods*, pages 155–160. Springer.

Martín-Fernández, J., Pawlowsky-Glahn, V., Egozcue, J., and Tolosona-Delgado, R. (2018). Advances in principal balances for compositional data. *Mathematical Geosciences*, **50**(3), 273–298.

Martín-Fernández, J. A., Hron, K., Templ, M., Filzmoser, P., and Palarea-Albaladejo, J. (2012). Model-based replacement of rounded zeros in compositional data: classical and robust approaches. *Computational Statistics & Data Analysis*, **56**(9), 2688–2704.

Martino, C., Morton, J. T., Marotz, C. A., Thompson, L. R., Tripathi, A., Knight, R., and Zengler, K. (2019). A novel sparse compositional technique reveals microbial perturbations. *MSystems*, **4**(1).

Mert, M. C., Filzmoser, P., and Hron, K. (2015). Sparse principal balances. *Statistical Modelling*, **15**(2), 159–174.

Morton, J. T., Aksenov, A. A., Nothias, L. F., Foulds, J. R., Quinn, R. A., Badri, M. H., Swenson, T. L., Van Goethem, M. W., Northen, T. R., Vazquez-Baeza, Y., *et al.* (2019). Learning representations of microbe–metabolite interactions. *Nature methods*, **16**(12), 1306–1314.

Noguera-Julian, M., Rocafort, M., Guillén, Y., Rivera, J., Casadellà, M., Nowak, P., Hildebrand, F., Zeller, G., Parera, M., Bellido, R., *et al.* (2016). Gut microbiota linked to sexual preference and hiv infection. *EBioMedicine*, **5**, 135–146.

Pawlowsky-Glahn, V., Egozcue, J. J., Tolosana Delgado, R., *et al.* (2011). Principal balances. *Proceedings of CoDaWork*, pages 1–10.

Quinn, T., Nguyen, D., Rana, S., Gupta, S., and Venkatesh, S. (2020). Deepcoda: personalized interpretability for compositional health data. In *International Conference on Machine Learning*, pages 7877–7886. PMLR.

Quinn, T. P. and Erb, I. (2020). Amalgams: data-driven amalgamation for the dimensionality reduction of compositional data. *NAR Genomics and Bioinformatics*, **2**(4), lqaa076.

Quinn, T. P., Gordon-Rodriguez, E., and Erb, I. (2021). A critique of differential abundance analysis, and advocacy for an alternative. *arXiv preprint arXiv:2104.07266*.

Rivera-Pinto, J., Egozcue, J. J., Pawlowsky-Glahn, V., Paredes, R., Noguera-Julian, M., and Calle, M. L. (2018). Balances: a new perspective for microbiome analysis. *MSystems*, **3**(4).

Susin, A., Wang, Y., Lê Cao, K.-A., and Calle, M. L. (2020). Variable selection in microbiome compositional data analysis. *NAR Genomics and Bioinformatics*, **2**(2), lqaa029.

Templ, M. (2020). Artificial neural networks to impute rounded zeros in compositional data. *arXiv preprint arXiv:2012.10300*.

Tolosana-Delgado, R., Talebi, H., Khodadadzadeh, M., and Van den Boogaart, K. (2019). On machine learning algorithms and compositional data. In *Proceedings of the 8th International Workshop on Compositional Data Analysis, Terrassa, Spain*, pages 3–8.

Tutz, G. and Gertheiss, J. (2016). Regularized regression for categorical data. *Statistical Modelling*, **16**(3), 161–200.

Wilcoxon, F. (1992). Individual comparisons by ranking methods. In *Breakthroughs in statistics*, pages 196–202. Springer.

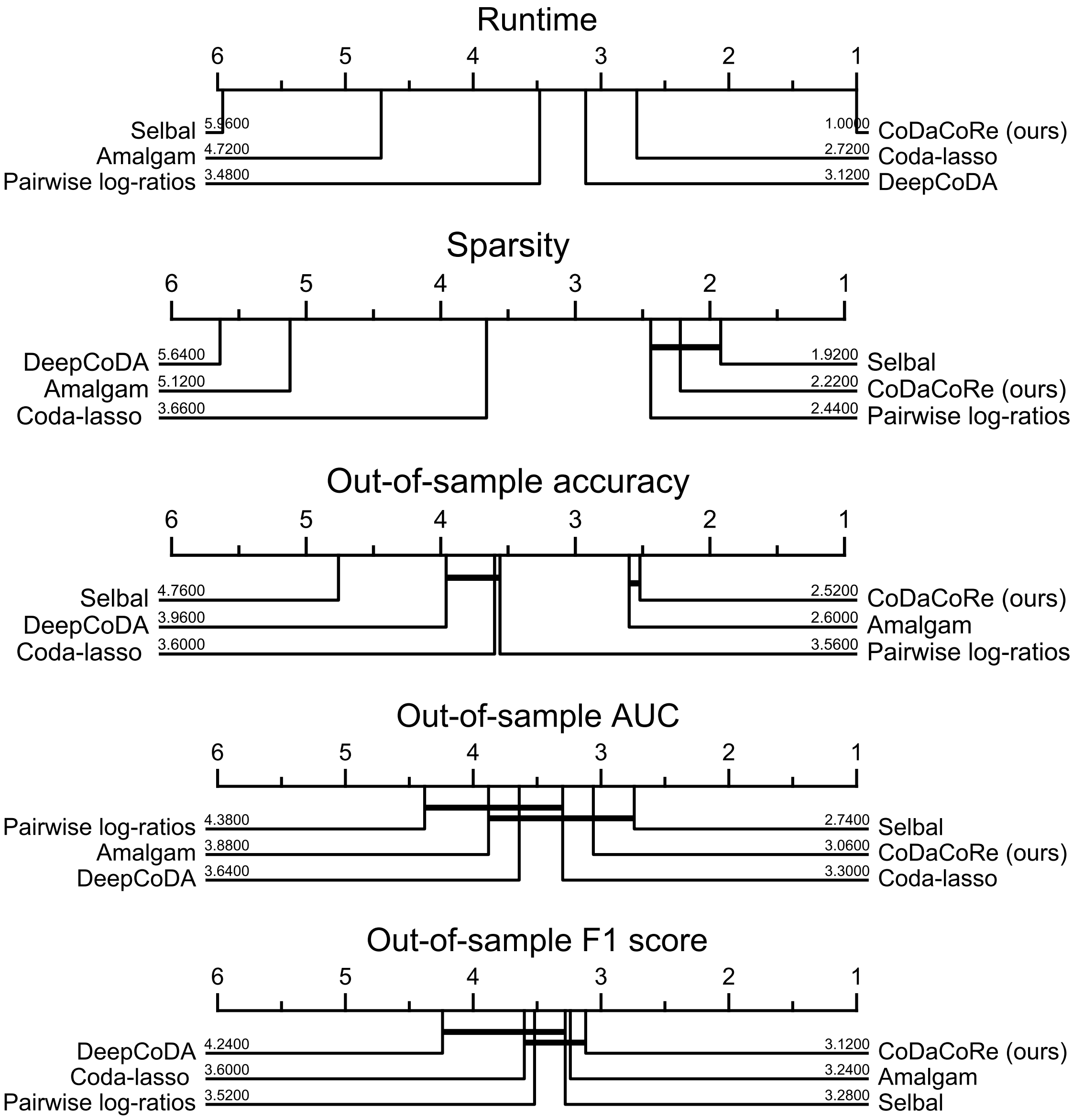

Figure 2: Critical difference diagrams for 5 different performance metrics, computed using the Wilcoxon-Holm method at 5% significance. CoDaCoRe is the most computationally efficient and the sparsest (tied with selbal and pairwise log-ratios), as well as being tied to the leading position in all 3 metrics of predictive accuracy.

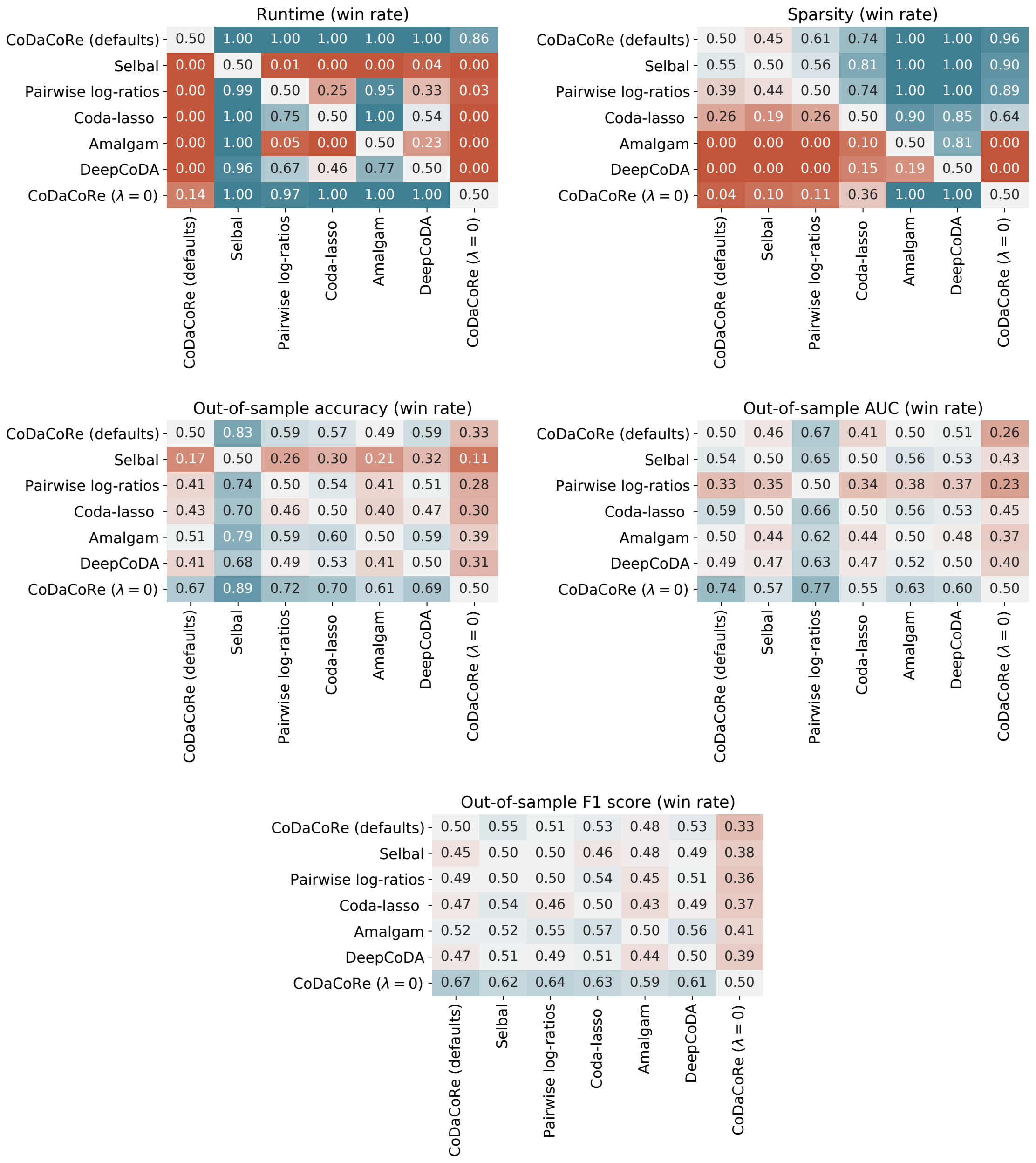

Figure 3: Pairwise win rates for each evaluation metric. The (i, j)th entry shows the proportion of runs in which the ith method achieved a better score than the jth method, where i indexes rows and j indexes columns. Note that, while CoDaCoRe (with default parameters) substantially outperforms in terms of runtime and sparsity, the only method that consistently outperforms in terms of predictive accuracy is CoDaCoRe with $\lambda = 0$.

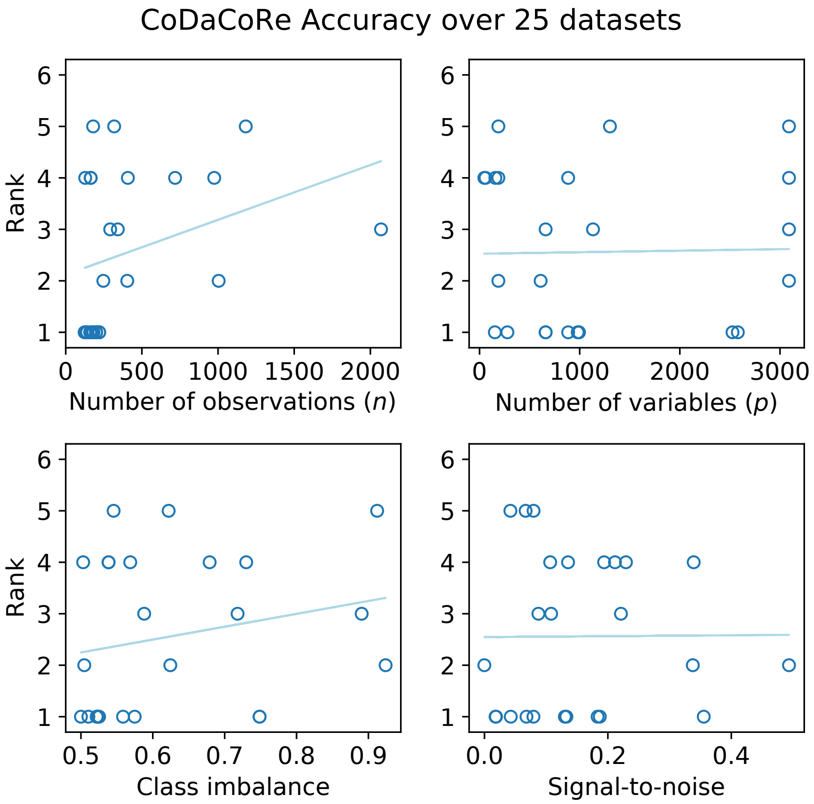

Figure 4: Rank scores of CoDaCoRe (with default parameters) relative to its competitors regressed against 4 dataset-level metrics of interest. Each point represents a different dataset, of which there are 25 total. Trendlines were obtained from 4 separate univariate least squares fits. Class imbalance is shown as a number between 0.5 and 1, denoting the proportion of datapoints in the majority class. Signal-to-noise ratio is estimated using the out-of-sample accuracy of the Random Forest model (note this is a rough proxy intended for illustrative purposes only). Note that CoDaCoRe tends to outperform the most on datasets with a low number of samples, and balanced classes.

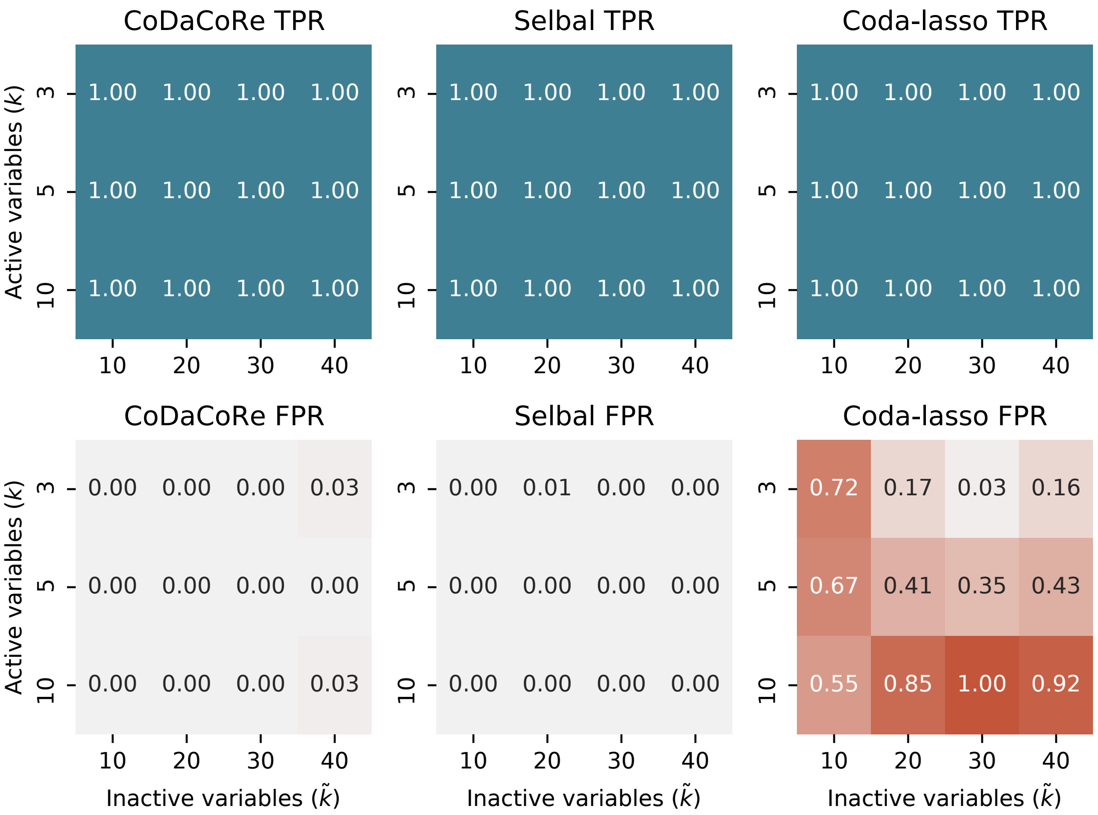

Figure 5: Variable selection performance for CoDaCoRe, selbal, and coda-lasso for a range of simulated datasets. True positive rates represent the proportion of active variables that were selected by the model, averaged over 10 independent draws of the synthetic dataset for each combination of k (active variables) and $\tilde{k}$ (inactive variables). False positive rates represent the proportion of inactive variables that were selected by the model, averaged over the same draws. In all, $3 \times 4 \times 10 = 120$ datasets were used to produce this table, and the response variables were simulated using balances.

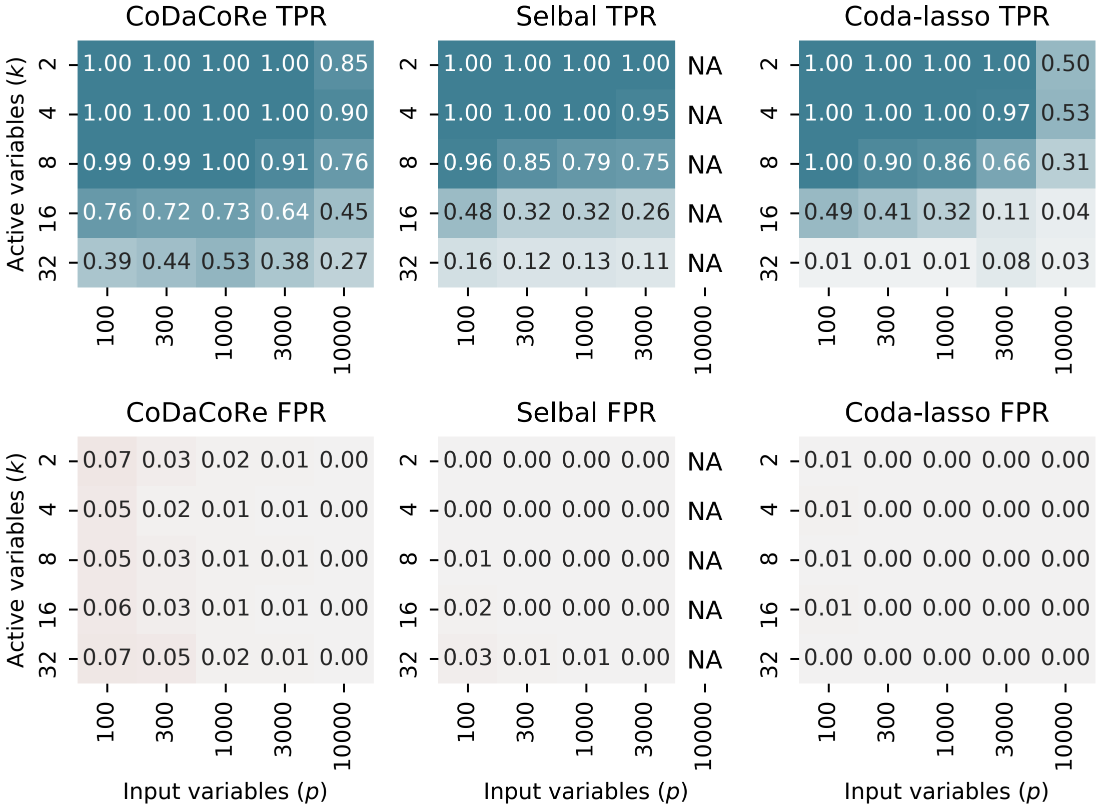

Figure 6: Variable selection performance for CoDaCoRe, selbal, and coda-lasso for a range of simulated datasets. True positive rates represent the proportion of active variables that were selected by the model, averaged over 10 independent draws of the synthetic dataset for each combination of k (active variables) and p (input variables). False positive rates represent the proportion of inactive variables that were selected by the model, averaged over the same draws. In all, $5 \times 5 \times 10 = 250$ datasets were used to produce this table, and the response variables were simulated using balances.

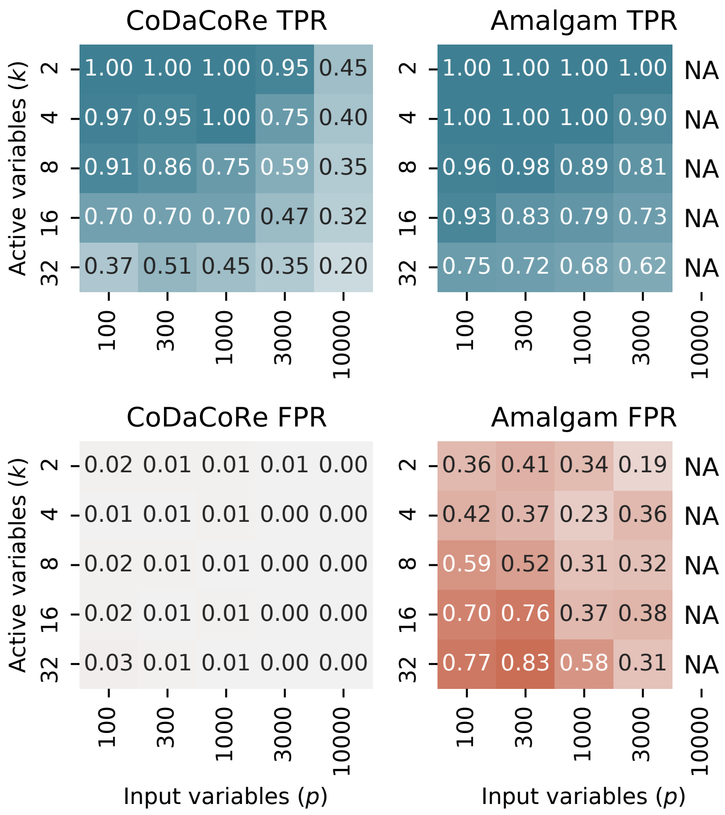

Figure 7: Variable selection performance for CoDaCoRe and amalgam, for a range of simulated datasets. True positive rates represent the proportion of active variables that were selected by the model, averaged over 10 independent draws of the synthetic dataset for each combination of k (active variables) and p (input variables). False positive rates represent the proportion of inactive variables that were selected by the model, averaged over the same draws. In all, $5 \times 5 \times 10 = 250$ datasets were used to produce this table, and the response variables were simulated using summed log-ratios.

Table 3: Data description. n denotes the number of observations, p the number of input variables. We also show the number of observations in the case and control groups.

Dataset ID	n	p	Group 1	Group 2
1	975	48	Crohn's disease	Without
2	128	60	Men who have sex with men	Without
3	220	153	Control	IBD
4	164	158	Crohn's disease	Ulcerative colitis
5	220	885	Control	IBD
6	164	885	Crohn's disease	Ulcerative colitis
7	182	278	Case	Diarrheal control
8	247	610	Case	Non-Diarrheal control
9	292	1133	Colorectal cancer (CRC)	Without
10	318	1302	Colorectal cancer (CRC)	Non-CRC control
11	1182	188	Primary solid tumor	Solid tissue normal
12	1004	188	Her2 Cancer	Not Her2 Cancer
13	718	188	LumA Cancer	LumB Cancer
14	140	992	Crohn's disease (ileum)	Without (ileum)
15	160	992	Crohn's disease (rectum)	Without (rectum)
16	2070	3090	GI tract	Oral
17	180	3090	Female	Male
18	404	3090	Stool	Tongue (dorsum)
19	408	3090	Subgingival plaque	Supragingival plaque
20	172	980	Healthy	Colorectal cancer
21	124	2526	Without	Diabetes
22	130	2579	Cirrhosis	Without
23	199	660	Black	Hispanic
24	342	660	Nugent score high	Nugent score low
25	200	660	Black	White

Table 4: Data description.

Dataset ID	Group 1 Size	Group 2 Size	Source
1	662	313	doi: 10.1016/j.chom.2014.02.005
2	73	55	doi: 10.1016/j.ebiom.2016.01.032
3	56	164	doi: 10.1038/s41564-018-0306-4
4	88	76	doi: 10.1038/s41564-018-0306-4
5	56	164	doi: 10.1038/s41564-018-0306-4
6	88	76	doi: 10.1038/s41564-018-0306-4
7	93	89	doi: 10.1128/mBio.01021-14
8	93	154	doi: 10.1128/mBio.01021-15
9	120	172	doi: 10.1186/s13073-016-0290-3
10	120	198	doi: 10.1186/s13073-016-0290-3
11	1078	104	doi: 10.1038/ng.2764
12	77	927	doi: 10.1038/ng.2764
13	524	194	doi: 10.1038/ng.2764
14	78	62	doi: 10.1016/j.chom.2014.02.005
15	68	92	doi: 10.1016/j.chom.2014.02.005
16	227	1843	doi: 10.1038/nature11209
17	82	98	doi: 10.1038/nature11209
18	204	200	doi: 10.1038/nature11209
19	203	205	doi: 10.1038/nature11209
20	86	86	doi: 10.1101/gr.126573.111
21	59	65	doi: 10.1038/nature11450
22	68	62	doi: 10.1038/nature13568
23	104	95	doi: 10.1073/pnas.1002611107
24	97	245	doi: 10.1073/pnas.1002611107
25	104	96	doi: 10.1073/pnas.1002611107

Table 5: Average runtime over 20 train/test splits, in seconds.

Dataset ID	CoDaCoRe (Defaults)	selbal	Pairwise log-ratios	Coda-lasso	amalgam	Random Forest
1	6	328	17	39	48	2
2	5	354	7	19	40	0
3	5	1307	50	62	145	1
4	4	1291	28	44	212	1
5	4	31407	638	420	2553	5
6	4	26638	387	348	3806	4
7	4	3316	79	39	532	1
8	4	14058	522	157	1965	3
9	4	48394	985	391	4694	11
10	5	60138	1540	505	5300	15
11	5	1954	240	90	315	5
12	6	2290	144	59	277	6
13	5	2014	117	107	307	5
14	4	33619	961	99	2919	2
15	5	35193	1326	116	2957	3
16	6	279072	298672	7776	40949	135
17	5	300431	10449	1628	22777	10
18	3	322726	9997	3131	25130	8
19	6	325642	24691	2890	24908	25
20	4	33150	468	208	2822	3
21	4	196216	1036	2942	15328	6
22	4	208464	769	4668	20612	6
23	4	15818	530	84	1447	2
24	4	16382	969	139	1621	4
25	4	15640	553	113	2349	2
Mean	5	79034	14207	1043	7361	11

Table 6: Proportion of input variables active (%), averaged over 20 train/test splits

Dataset ID	CoDaCoRe (Defaults)	selbal	Pairwise log-ratios	Coda-lasso	amalgam	Random Forest
1	15.4±2.4	25.6±3.1	21.0±2.4	53.8±5.5	85.2±2.8	100.0±0.0
2	4.5±0.6	5.5±0.4	11.2±1.9	9.4±0.7	86.1±2.2	100.0±0.0
3	2.1±0.3	3.0±0.5	3.5±0.9	6.7±1.6	80.7±2.7	100.0±0.0
4	2.0±0.3	1.8±0.2	4.2±0.8	6.3±1.4	81.6±1.6	100.0±0.0
5	0.6±0.3	0.2±0.0	0.7±0.1	1.9±0.7	84.9±0.9	100.0±0.0
6	0.2±0.0	0.3±0.0	0.8±0.1	0.7±0.1	85.3±0.8	100.0±0.0
7	3.3±0.6	0.8±0.1	0.2±0.1	2.2±0.5	79.6±2.5	100.0±0.0
8	1.3±0.2	1.0±0.1	1.7±0.2	4.2±1.0	81.9±2.2	100.0±0.0
9	0.8±0.1	0.2±0.0	0.2±0.1	1.2±0.4	89.7±1.0	100.0±0.0
10	0.6±0.1	0.2±0.0	0.0±0.0	1.1±0.2	90.0±1.3	100.0±0.0
11	1.8±0.9	4.2±0.5	2.5±0.3	74.2±10.6	91.0±1.7	100.0±0.0
12	5.3±0.9	6.9±0.5	5.0±0.9	62.1±8.8	89.0±1.5	100.0±0.0
13	5.2±0.4	7.1±0.4	5.9±0.8	58.1±12.7	84.5±1.7	100.0±0.0
14	0.8±0.2	0.2±0.0	0.6±0.1	0.4±0.1	92.9±1.4	100.0±0.0
15	0.6±0.2	0.3±0.1	0.6±0.1	0.6±0.2	90.8±1.1	100.0±0.0
16	0.1±0.0	0.4±0.0	0.2±0.0	95.1±9.9	94.8±1.3	100.0±0.0
17	0.1±0.0	0.1±0.0	0.0±0.0	0.0±0.0	94.0±2.6	100.0±0.0
18	0.1±0.0	0.3±0.0	0.3±0.0	100.0±0.0	88.6±4.5	100.0±0.0
19	0.1±0.0	0.1±0.0	0.3±0.0	0.4±0.1	97.8±1.1	100.0±0.0
20	0.5±0.1	0.2±0.0	0.4±0.1	0.4±0.5	89.1±2.5	100.0±0.0
21	0.3±0.1	0.1±0.0	0.0±0.0	1.1±0.9	92.1±3.7	100.0±0.0
22	0.1±0.0	0.1±0.0	0.3±0.1	10.0±10.7	89.9±1.8	100.0±0.0
23	0.5±0.1	0.3±0.0	0.2±0.1	0.5±0.3	86.7±3.3	100.0±0.0
24	1.0±0.1	0.8±0.1	1.9±0.1	1.6±0.1	93.2±1.2	100.0±0.0
25	0.3±0.0	0.3±0.0	0.7±0.1	0.2±0.1	69.8±5.7	100.0±0.0
Mean	1.9±0.3	2.4±0.2	2.5±0.4	19.7±2.7	87.6±2.1	100.0±0.0

Table 7: Out-of-sample accuracy (%) averaged over 20 train/test splits.

Dataset ID	CoDaCoRe (Defaults)	selbal	Pairwise log-ratios	Coda-lasso	amalgam	Random Forest
1	71.4±1.1	45.4±2.9	68.5±1.0	72.4±1.7	76.1±1.4	81.5±1.4
2	87.5±2.5	60.6±3.6	88.3±3.1	88.7±2.8	89.4±2.8	90.8±2.3
3	76.7±2.4	73.3±0.0	73.4±0.2	66.0±3.0	73.7±2.4	81.7±1.5
4	61.9±2.6	52.6±1.9	70.0±2.7	64.8±3.0	67.8±3.7	75.0±2.4
5	85.0±2.0	73.4±0.2	82.4±1.5	76.6±3.4	82.7±2.4	87.9±2.0
6	65.3±3.5	55.1±2.0	68.2±3.0	67.3±3.0	63.1±2.9	73.2±3.1
7	69.0±3.8	64.0±2.8	53.0±2.0	66.9±4.0	60.4±2.9	69.7±5.6
8	92.9±1.4	69.2±1.8	88.6±2.3	93.1±1.4	92.1±1.5	96.2±1.0
9	61.4±2.9	64.2±1.4	59.1±0.3	59.1±3.9	65.7±3.2	67.5±2.1
10	63.1±1.6	66.0±0.7	62.5±0.0	65.9±3.3	63.2±2.7	66.4±1.1
11	96.8±0.5	68.1±9.2	98.9±0.3	97.3±0.6	99.1±0.2	99.2±0.2
12	92.9±0.5	92.1±0.0	92.2±0.2	85.8±1.0	92.6±0.6	92.4±0.2
13	79.1±1.0	27.2±0.1	75.5±0.7	80.5±1.2	82.6±1.0	83.7±1.2
14	68.5±3.8	50.3±1.8	68.1±3.8	62.2±4.0	65.0±3.5	63.8±4.3
15	74.5±4.9	57.9±1.8	72.0±3.3	71.5±4.8	65.8±3.2	70.8±5.0
16	99.9±0.1	88.9±0.0	100.0±0.0	99.9±0.1	99.9±0.1	99.9±0.1
17	51.4±3.3	54.8±1.7	53.5±0.7	46.0±0.0	51.9±4.0	61.2±2.9
18	100.0±0.0	49.9±0.5	100.0±0.0	100.0±0.0	99.5±0.5	99.8±0.4
19	64.7±2.5	55.2±1.5	65.2±2.0	70.5±1.8	70.8±1.6	73.2±2.1
20	69.0±4.1	62.4±2.2	66.7±3.6	52.6±2.4	60.6±3.6	68.3±3.0
21	60.8±4.1	54.6±2.6	52.2±0.4	54.8±4.9	57.4±6.1	54.4±5.4
22	77.2±3.2	64.6±4.0	75.9±5.5	72.9±3.9	74.1±3.6	88.0±2.1
23	59.0±3.4	48.9±1.5	52.0±1.8	52.5±1.3	56.2±3.0	54.0±3.0
24	93.3±1.4	76.5±1.6	92.9±1.3	93.8±1.3	93.6±0.8	93.9±1.1
25	59.6±3.1	53.9±1.7	53.7±2.9	51.2±0.0	57.7±3.7	56.5±2.4
Mean	75.2±2.4	61.2±1.9	73.3±1.7	72.5±2.3	74.4±2.5	78.0±2.2

Table 8: Out-of-sample AUC (%) averaged over 20 train/test splits.

Dataset ID	CoDaCoRe (Defaults)	selbal	Pairwise log-ratios	Coda-lasso	amalgam	Random Forest
1	75.8±1.7	81.5±1.5	69.9±2.5	81.2±1.8	81.0±1.5	87.8±1.2
2	93.9±1.9	92.3±2.1	94.2±2.0	94.8±1.7	94.7±2.1	96.2±2.0
3	79.2±2.9	76.0±3.2	77.3±3.2	73.9±5.5	76.9±3.9	88.1±1.6
4	69.4±3.3	73.4±4.5	74.3±3.0	74.0±3.3	72.6±3.4	83.2±2.8
5	91.2±1.6	93.1±1.1	90.0±1.8	92.1±1.8	88.8±2.8	93.7±1.3
6	72.0±3.7	74.9±4.1	76.2±3.7	76.0±3.7	68.0±3.5	81.8±3.3
7	73.7±4.0	82.1±3.0	53.0±2.9	75.6±4.9	65.8±3.5	75.3±4.7
8	98.2±0.7	96.5±0.9	94.8±2.0	98.8±0.5	97.6±0.8	99.3±0.3
9	64.6±3.0	61.6±3.5	51.5±1.6	61.9±4.3	67.7±3.5	70.5±2.1
10	66.2±3.7	62.8±2.0	50.0±0.0	68.5±2.7	64.3±3.1	66.0±2.0
11	98.5±0.5	99.6±0.3	99.5±0.4	99.4±0.6	99.4±0.5	99.3±0.7
12	89.2±1.6	91.1±1.5	88.1±1.6	94.4±1.2	90.0±1.6	90.1±1.3
13	82.5±1.4	90.6±1.2	78.0±1.7	88.4±1.2	87.1±1.9	89.7±1.2
14	73.5±4.3	68.8±4.7	70.8±5.1	63.4±5.8	68.8±4.7	71.3±5.1
15	79.9±5.5	79.2±3.7	79.0±3.5	75.4±6.2	69.0±3.6	78.4±4.6
16	100.0±0.0	100.0±0.0	100.0±0.0	100.0±0.0	100.0±0.0	100.0±0.0
17	57.2±2.5	54.2±3.5	50.6±1.7	50.0±0.0	56.1±4.0	65.1±2.8
18	100.0±0.0	100.0±0.0	100.0±0.0	100.0±0.0	99.8±0.3	100.0±0.0
19	71.6±3.6	80.0±1.6	70.2±2.4	79.3±1.6	76.2±1.6	79.8±2.1
20	75.0±4.0	76.9±3.0	70.2±4.8	53.7±3.4	65.1±3.0	72.9±3.0
21	67.7±4.9	62.2±3.8	50.6±1.2	61.2±5.8	67.2±5.4	61.3±4.6
22	85.8±2.6	85.0±2.4	82.0±6.4	89.2±2.1	81.8±3.8	91.8±2.3
23	64.6±3.6	57.3±3.2	52.3±2.8	52.6±2.4	61.7±3.4	57.3±3.1
24	96.2±1.3	97.2±0.8	96.0±1.1	97.5±0.7	96.4±1.5	98.1±0.6
25	62.7±3.8	63.5±3.2	60.8±3.4	49.9±0.1	59.4±3.6	58.2±2.6
Mean	79.5±2.6	80.0±2.4	75.2±2.4	78.0±2.4	78.2±2.7	82.2±2.2

Table 9: Out-of-sample F1 score (%) averaged over 20 train/test splits.

Dataset ID	CoDaCoRe (Defaults)	selbal	Pairwise log-ratios	Coda-lasso	amalgam	Random Forest
1	46.9±2.8	53.8±1.2	10.9±6.5	65.3±1.8	58.6±2.6	65.7±2.8
2	85.6±2.6	68.0±2.1	86.0±3.6	86.4±3.8	87.1±3.6	89.1±2.8
3	84.1±1.8	84.6±0.0	84.6±0.1	73.3±2.9	82.2±1.6	88.4±0.9
4	56.8±3.6	65.7±1.1	65.0±3.1	65.0±3.6	66.4±4.0	71.7±3.0
5	89.6±1.4	84.7±0.1	88.7±0.9	81.0±3.5	87.9±1.7	91.8±1.4
6	60.9±4.5	66.2±1.6	63.6±4.6	66.2±3.5	56.9±4.5	70.4±3.9
7	69.0±3.9	72.9±1.7	10.6±9.5	70.0±7.8	60.3±3.6	69.3±5.2
8	94.3±1.2	80.2±0.9	91.2±1.7	94.6±1.0	93.7±1.2	97.0±0.8
9	68.3±2.5	76.8±0.7	74.2±0.3	62.2±10.2	70.9±2.9	76.6±1.6
10	72.1±1.3	78.6±0.4	76.9±0.0	73.6±4.0	70.6±2.4	77.9±0.7
11	80.8±2.8	38.9±3.9	93.5±1.8	87.0±2.5	94.9±1.3	95.2±1.4
12	96.2±0.3	95.9±0.0	95.9±0.1	91.8±0.7	96.1±0.3	96.0±0.1
13	54.7±2.1	42.7±0.1	31.6±3.9	68.2±1.9	64.5±2.8	62.7±3.5
14	63.2±3.9	64.1±1.1	52.9±9.8	38.1±13.7	60.5±4.3	53.0±6.9
15	77.5±4.4	72.5±1.2	75.9±3.0	75.0±4.7	69.8±3.5	75.9±4.0
16	100.0±0.0	94.1±0.0	100.0±0.0	100.0±0.0	100.0±0.0	99.9±0.1
17	58.4±3.6	69.8±1.2	69.4±1.1	0.0±0.0	57.0±4.2	65.8±2.9
18	100.0±0.0	66.3±0.2	100.0±0.0	100.0±0.0	99.5±0.5	99.8±0.4
19	64.5±2.5	69.0±0.7	65.7±2.1	73.0±1.8	71.3±1.7	73.4±2.3
20	69.5±3.6	71.9±1.2	68.9±2.6	14.8±11.9	60.5±3.6	66.9±3.1
21	62.1±4.5	67.1±2.5	68.2±0.4	34.1±14.8	59.3±5.9	55.8±6.1
22	76.8±3.1	72.7±2.2	74.4±6.4	77.4±2.6	73.2±4.1	88.1±1.9
23	60.8±5.0	64.1±1.0	7.3±4.8	10.2±7.8	51.6±3.6	50.4±3.3
24	95.4±0.9	85.8±0.8	95.2±0.9	95.8±0.8	95.6±0.6	95.7±0.8
25	54.7±3.8	66.8±1.0	43.0±8.0	2.5±5.0	59.7±4.2	55.5±2.9
Mean	73.7±2.6	70.9±1.1	67.8±3.0	64.2±4.4	73.9±2.8	77.3±2.5
