# Supplementary material for: Learning sparse log-ratios for high-throughput sequencing data
Source: Bioinformatics. 2021 Sep 8;38(1):157–63. doi: 10.1093/bioinformatics/btab645 (PMC8696089; doi:10.1093/bioinformatics/btab645)
Supplement: btab645_Supplementary_Data [file btab645_supplementary_data.zip › balancesSelection.pdf]

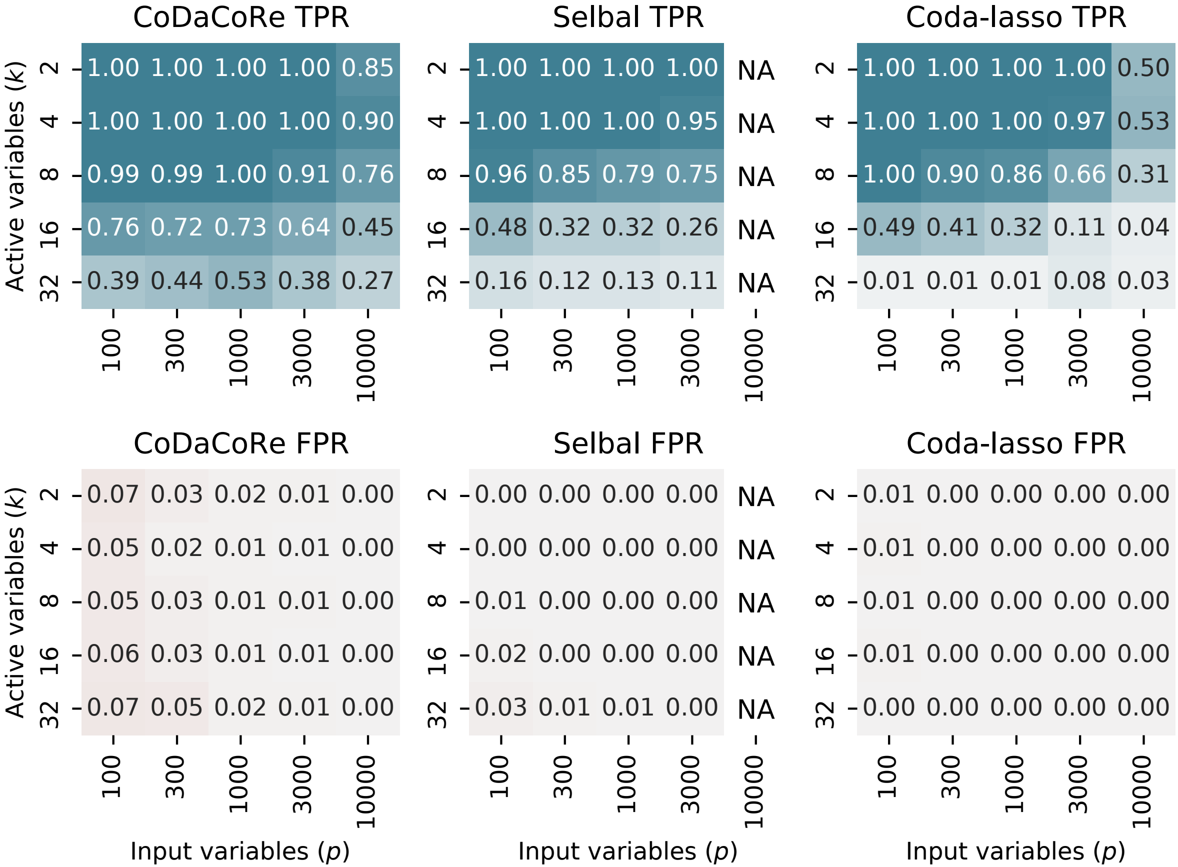
CoDaCoRe TPR
Selbal TPR
Coda-lasso TPR
CoDaCoRe FPR
Selbal FPR
Coda-lasso FPR
Active variables (k)
Input variables (p)
2
4
8
16
32
100
300
1000
3000
10000
1.00 1.00 1.00 1.00 0.85
1.00 1.00 1.00 1.00 0.90
0.99 0.99 1.00 0.91 0.76
0.76 0.72 0.73 0.64 0.45
0.39 0.44 0.53 0.38 0.27
1.00 1.00 1.00 1.00 NA
1.00 1.00 1.00 0.95 NA
0.96 0.85 0.79 0.75 NA
0.48 0.32 0.32 0.26 NA
0.16 0.12 0.13 0.11 NA
1.00 1.00 1.00 1.00 0.50
1.00 1.00 1.00 0.97 0.53
1.00 0.90 0.86 0.66 0.31
0.49 0.41 0.32 0.11 0.04
0.01 0.01 0.01 0.08 0.03
0.07 0.03 0.02 0.01 0.00
0.05 0.02 0.01 0.01 0.00
0.05 0.03 0.01 0.01 0.00
0.06 0.03 0.01 0.01 0.00
0.07 0.05 0.02 0.01 0.00
0.00 0.00 0.00 0.00 NA
0.00 0.00 0.00 0.00 NA
0.01 0.00 0.00 0.00 NA
0.02 0.00 0.00 0.00 NA
0.03 0.01 0.01 0.00 NA
0.01 0.00 0.00 0.00 0.00
0.01 0.00 0.00 0.00 0.00
0.01 0.00 0.00 0.00 0.00
0.01 0.00 0.00 0.00 0.00
0.00 0.00 0.00 0.00 0.00
