# Supplementary figures and images for: Learning sparse log-ratios for high-throughput sequencing data
Source: Bioinformatics. 2021 Sep 8;38(1):157–63. doi: 10.1093/bioinformatics/btab645 (PMC8696089; doi:10.1093/bioinformatics/btab645)

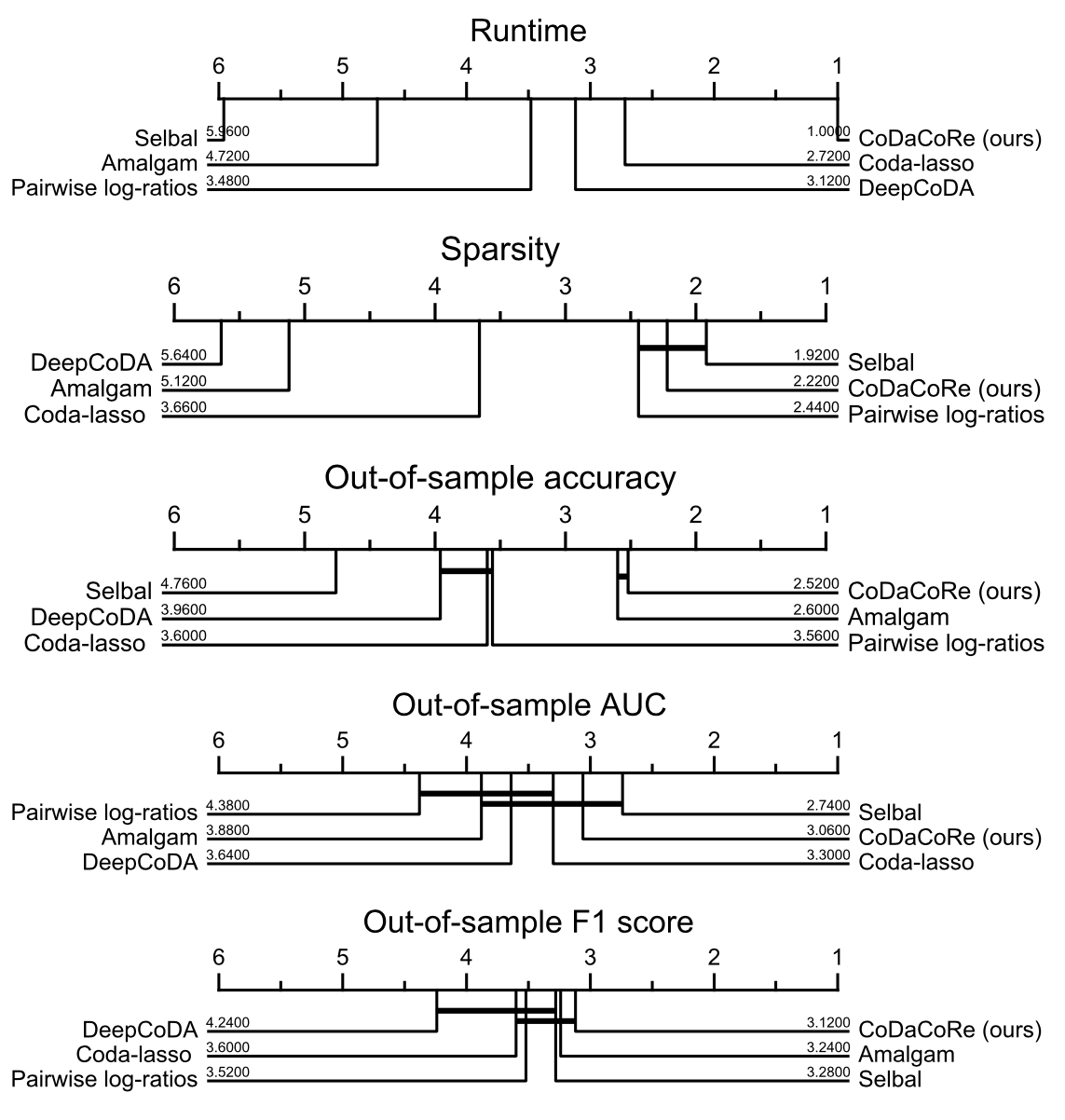

Supplement: btab645_Supplementary_Data [file btab645_supplementary_data.zip › Figure2.png]

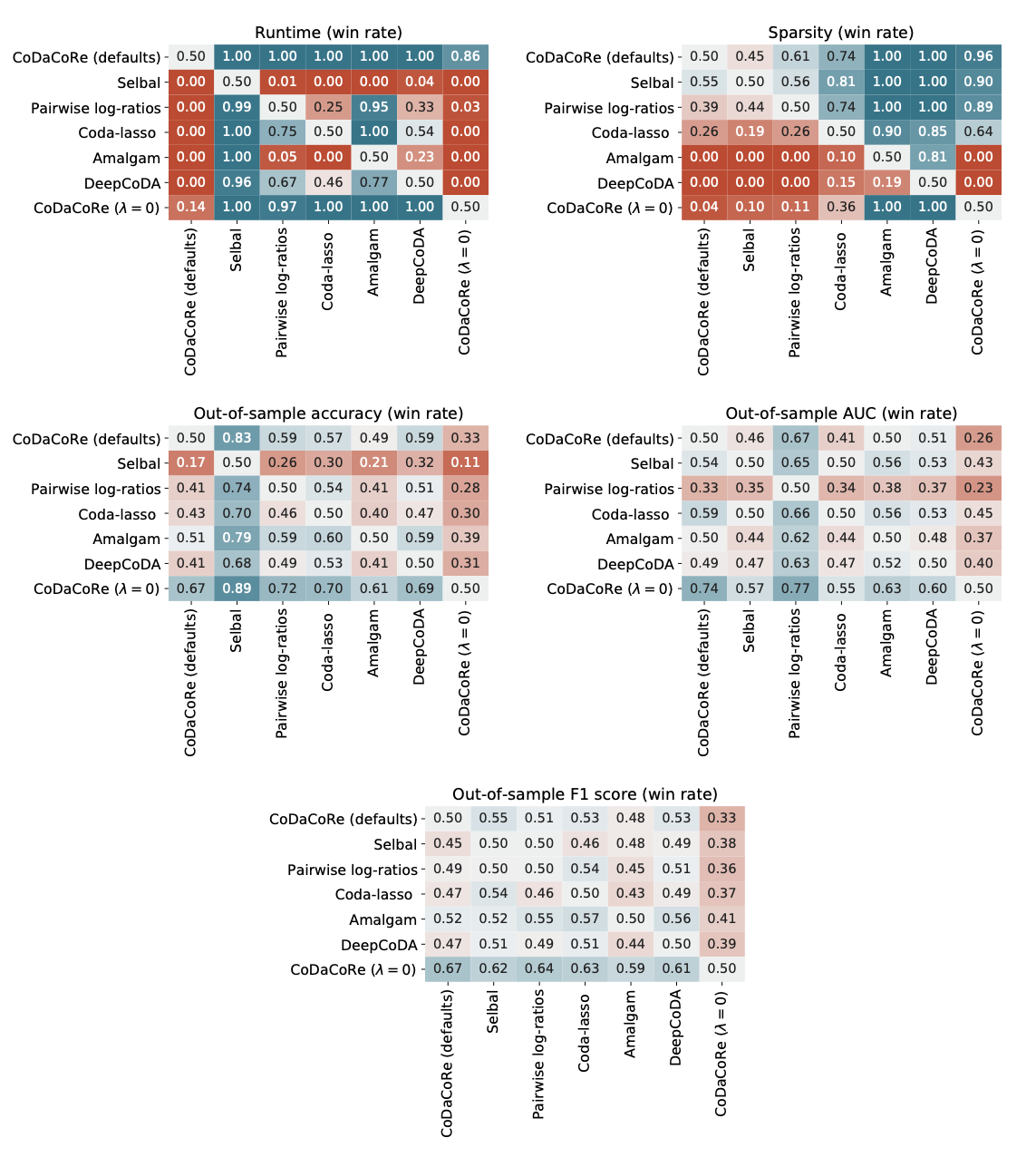

Supplement: btab645_Supplementary_Data [file btab645_supplementary_data.zip › Figure3.png]

# CoDaCoRe Accuracy over 25 datasets

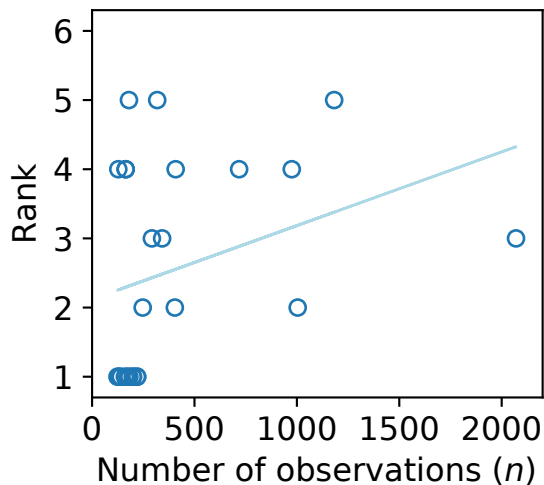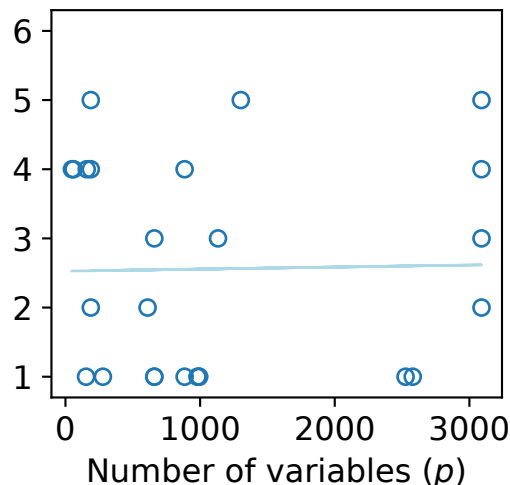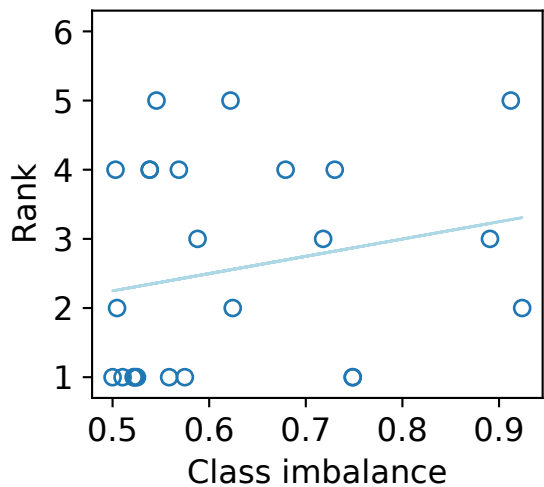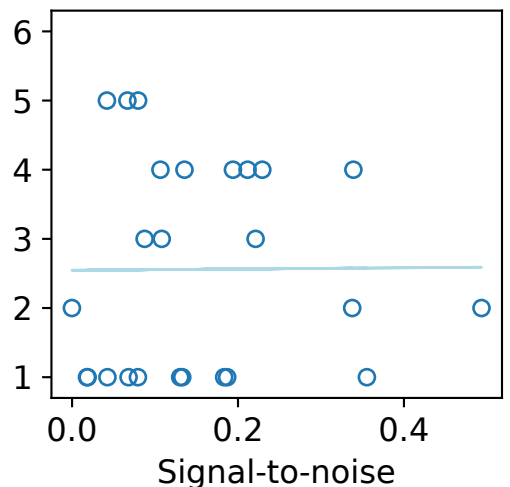

Supplement: btab645_Supplementary_Data [file btab645_supplementary_data.zip › ablations.pdf]

CoDaCoRe TPR

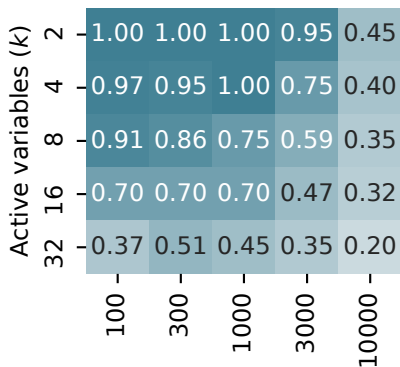

Amalgam TPR

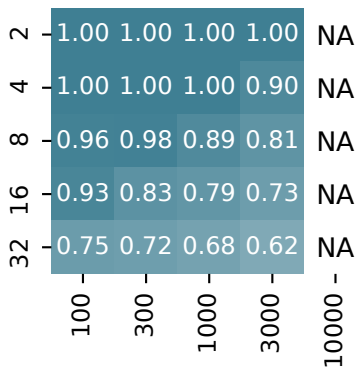

CoDaCoRe FPR

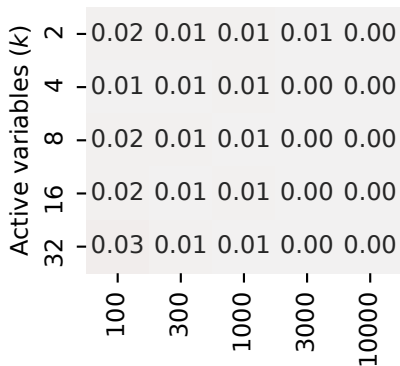

Amalgam FPR

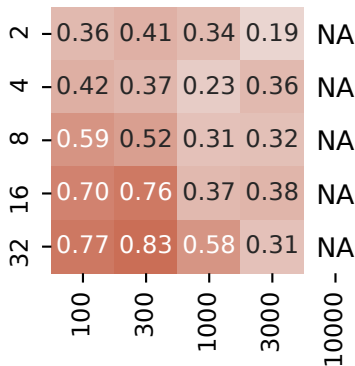Input variables ( $p$ )Input variables ( $p$ )

Supplement: btab645_Supplementary_Data [file btab645_supplementary_data.zip › amalgamSelection.pdf]

CoDaCoRe TPR

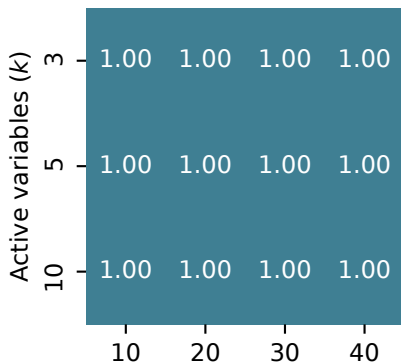

Selbal TPR

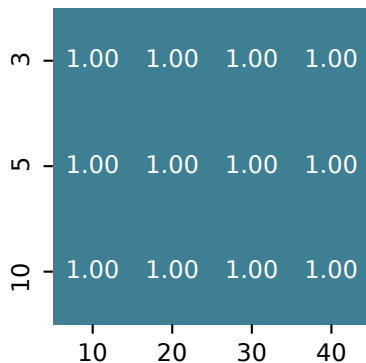

Coda-lasso TPR

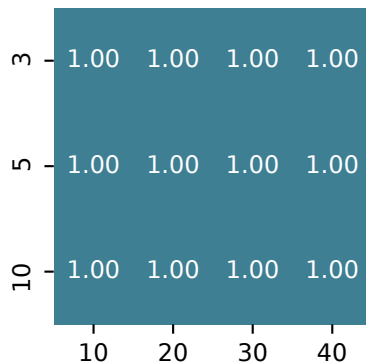

CoDaCoRe FPR

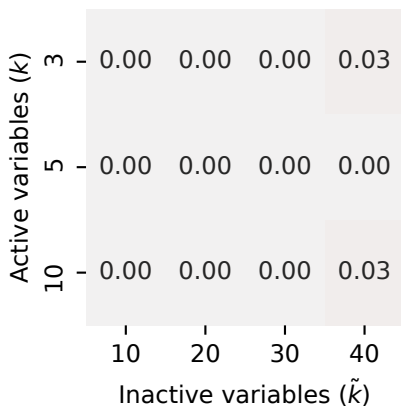

Selbal FPR

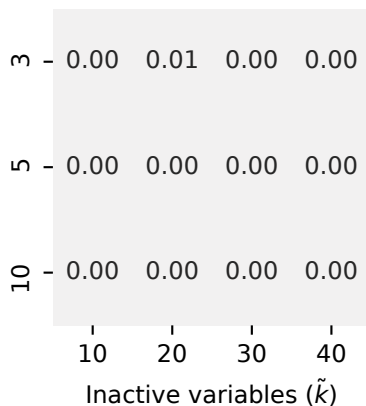

Coda-lasso FPR

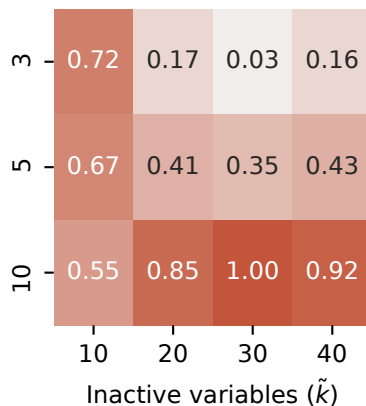

Supplement: btab645_Supplementary_Data [file btab645_supplementary_data.zip › balancesSelectionSusin.pdf]
